# Inhibitory effect of maple syrup on the cell growth and invasion of human colorectal cancer cells

**DOI:** 10.3892/or.2015.3777

**Published:** 2015-02-02

**Authors:** TETSUSHI YAMAMOTO, KENTARO UEMURA, KAHO MORIYAMA, KUNIKO MITAMURA, ATSUSHI TAGA

**Affiliations:** Pathological and Biomolecule Analyses Laboratory, Faculty of Pharmacy, Kinki University, Higashi-Osaka, Osaka 577-8502, Japan

**Keywords:** colon cancer, cell proliferation, cell invasion, AKT, phytomedicine

## Abstract

Maple syrup is a natural sweetener consumed by individuals of all ages throughout the world. Maple syrup contains not only carbohydrates such as sucrose but also various components such as organic acids, amino acids, vitamins and phenolic compounds. Recent studies have shown that these phenolic compounds in maple syrup may possess various activities such as decreasing the blood glucose level and an anticancer effect. In this study, we examined the effect of three types of maple syrup, classified by color, on the cell proliferation, migration and invasion of colorectal cancer (CRC) cells in order to investigate whether the maple syrup is suitable as a phytomedicine for cancer treatment. CRC cells that were administered maple syrup showed significantly lower growth rates than cells that were administered sucrose. In addition, administration of maple syrup to CRC cells caused inhibition of cell invasion, while there was no effect on cell migration. Administration of maple syrup clearly inhibited AKT phosphorylation, while there was no effect on ERK phosphorylation. These data suggest that maple syrup might inhibit cell proliferation and invasion through suppression of AKT activation and be suitable as a phytomedicine for CRC treatment, with fewer adverse effects than traditional chemotherapy.

## Introduction

Colorectal cancer (CRC) is one of the most common types of cancers and is the leading cause of cancer-related death worldwide (1). When the tumor is limited to the mucosa or submucosa, CRC can be completely cured by endoscopic or surgical therapy; however, many patients are already at an advanced stage at diagnosis. A chemotherapy regimen of fluoropyrimidines plus either oxaliplatin or irinotecan is considered the standard treatment for advanced CRC (2–5). Recently, interest in the use of phytomedicines, such as botanical extracts, for cancer treatment is increasing (6). These natural therapies using plant-derived extracts may reduce adverse side effects compared with traditional cancer treatments (7).

Maple syrup is a natural sweetener consumed by men and women of all ages throughout the world. Maple syrup contains not only abundant amounts of sucrose and glucose, but also various other components such as oligosaccharides, organic acids, amino acids, vitamins, and minerals including manganese and zinc (8–12). Moreover, recent studies have shown that maple syrup contains various phenolic compounds such as lignans and coumarin (13,14), quebecol (15), and ginnalin (16,17). These phenolic compounds in maple syrup may possess various types of activity. An *in vitro* study of a butanol extract from maple syrup demonstrated inhibitory activity toward α-glucosidase (18). Ethyl acetate extracts of maple syrup showed antioxidant activity and anti-proliferative effects against cancer cell lines (19). Ginnalin-A inhibited the cell growth of colon cancer cell lines (16). In addition, an effect of maple syrup has also been reported. The increase in plasma glucose was found to be lower after the oral administration of maple syrup than after the administration of sucrose in the Otsuka Long-Evans Tokushima fatty rat, a model of type II diabetes mellitus (20).

Although maple syrup is made from boiling down sap, its color, aroma and taste change due to differences in the growth conditions and season when the sap is collected. Thus, based on Canadian standards, maple syrup is classified into five grades as follows: AA (extra light), grade A (light), grade B (medium), grade C (amber), and grade D (dark) (19). The syrup typically becomes darker in color as the season progresses, and antioxidant activity is proportional to the darkening color of the maple syrup (19). This suggests that different grades of maple syrup may have different effects against cancer cells. However, there are almost no reports or scientific evidence on the effects of the various maple syrup grades on the behavior of cancer cells. Therefore, there is a need to evaluate differences in maple syrup grade on the functions of cancer cells to evaluate the use of maple syrup as a phytomedicine for cancer treatment. In this study, we examined the effect of three types of maple syrup, classified by color, on the proliferation, migration, and invasion of CRC cells in order to investigate whether maple syrup is suitable as a phytomedicine for cancer treatment.

## Materials and methods

### Materials

The following chemicals and reagents of the highest grade available were purchased as follows: urea from GE Healthcare, UK, Ltd. (Buckinghamshire, UK); 3-[(3-chol-amidepropyl)dimethylammonio]-1-propanesulphonate (CHAPS) from Wako Pure Chemical Industries (Osaka, Japan); and thiourea and Triton X-100 from Nacalai Tesque, Inc. (Kyoto, Japan). All other chemicals and reagents were purchased from Sigma Chemical Corp. (St. Louis, MO, USA).

### Maple syrup samples

Maple syrups were purchased at a local grocery store. We chose three maple syrups of different colors, and classified these syrups as three types based on their increasingly darker color. Maple syrup I was slightly golden, maple syrup II was amber; and maple syrup III was very dark brown.

### High-performance liquid chromatography (HPLC) analysis

We determined the sucrose concentration in each type of maple syrup using an LC-10Advp HPLC system equipped with Shodex RI-71 and an Asahipak NH2P-50 4E (all from Shimadzu Kyoto, Japan) column at room temperature. The mobile phase used was acetonitrile/milliQ water, 3:1 (v/v), at a flow rate of 1 ml/min, and a 20-μl sample solution was injected. The sample solution was prepared by diluting the syrup in water (1;100).

### CRC cell lines

The DLD-1 and SW480 colorectal cancer cell lines and CCD 841 CoN normal human colon epithelial cells were purchased from the American Type Culture Collection (ATTC; Manassas, VA, USA). All cells were cultured in RPMI-1640 medium supplemented with 10% fetal bovine serum (Gibco, Carlsbad, CA, USA) in an atmosphere containing 5% CO_2_.

### Cell proliferation assays

Cells were grown in 96-well plates at a density of 5×10^3^ cells per well and grown in culture medium. The next day, the medium was changed and cells were either grown in culture medium containing sucrose or maple syrup. After 24, 48, 72 and 96 h, the cells were incubated with WST-8 cell counting reagent (Wako) for 4 h at 37°C, and the optical density of the culture solution in the plate was measured using an ELISA plate reader. In addition to the WST-8 assay, the cell number was counted using a Countess Automated Cell Counter (Life Technologies Japan, Tokyo, Japan). The cells were cultured in a 6-well plate at a density of 5×10^4^ cells per well and grown in culture medium. On the next day, the medium was changed, and the cells were grown in culture medium containing either sucrose or maple syrup. The number of cells was counted after 24, 48, 72 and 96 h.

### Cell migration and invasion assays

The *in vitro* migration assay was carried out using a modified Boyden chamber technique (BD Bioscience, Franklin Lakes, NJ, USA). Cells were plated on the inner surface of the inserts at a density of 1×10^5^ cells/insert, followed by incubation at 37°C in a humidified 5% CO_2_ atmosphere. Culture medium containing sucrose or maple syrup was placed in each lower chamber as a chemoattractant. Using a previously reported method, the DLD-1 and SW480 cells on the outer surface of the inserts were counted after 48 (21) and 20 h (22), respectively. All assays were performed in triplicate, and 5 fields (x200) were counted on each membrane in a blinded manner. The cell invasion assay was performed using a modified Boyden chamber method in which the inner surfaces of the inserts were coated with Matrigel.

### Protein preparation

DLD-1 and SW480 cells were plated at a density of 5×10^5^ cells per 100-mm dish and grown in culture medium. On the following day, the medium was changed and cells were grown in culture medium containing either sucrose or maple syrup. After 72 h, the cells were solubilized in urea lysis buffer (7 M urea, 2 M thiourea, 5% CHAPS, 1% Triton X-100). The protein concentration was measured using the Bradford method.

### Western blot analysis

The cell extract was subjected to SDS-PAGE under reducing conditions, and the separated proteins were transferred to polyvinylidene fluoride transfer membranes. The membranes were incubated with an anti-phospho-p44/42 MAPK antibody or anti-phospho-AKT antibody (Cell Signaling Technology Inc., Beverly, MA, USA) at 4°C overnight. The membranes were washed and incubated with HRP-conjugated anti-rabbit IgG antibody or HRP-conjugated anti-mouse IgG antibody (American Qualex, San Clemente, CA, USA). After washing, the blots were visualized by enhanced chemiluminescence and detected using an ImageQuant LAS 500 system (GE Healthcare). The same membranes were re-probed with the anti-β-actin antibody (Sigma Chemical Corp., St. Louis, MO, USA), anti-p44/42 MAPK (Erk1/2) antibody, or anti-AKT antibody (Cell Signaling Technology) to confirm equal loading of the proteins. All western blot analyses were performed in triplicate.

### Statistical analysis

All data are presented as the mean ± standard error of measurement (SEM). The data were analyzed using one-way analysis of variance followed by Dunnett’s test. A P-value ≤0.05 was considered to indicate a statistically significant difference. Computations were performed using the GraphPad Prism version 5 (GraphPad Software, La Jolla, CA, USA).

## Results

### The sucrose concentration in the three types of maple syrup

First, we examined the concentration of sucrose, the main component of maple syrup (23), in the three selected maple syrups (Table I). The sucrose concentration was well controlled and the concentrations did not differ significantly among the three types of maple syrup. We accurately adjusted the concentration of sucrose in maple syrups II and III to those of maple syrup I to ensure that the cells were affected by an equivalent amount of sucrose in each dose of maple syrup. We also prepared a sucrose solution containing an equivalent amount of sucrose as in maple syrup I as a control solution.

### Cytotoxicity of sucrose against the CRC cells

**Figure 1:**
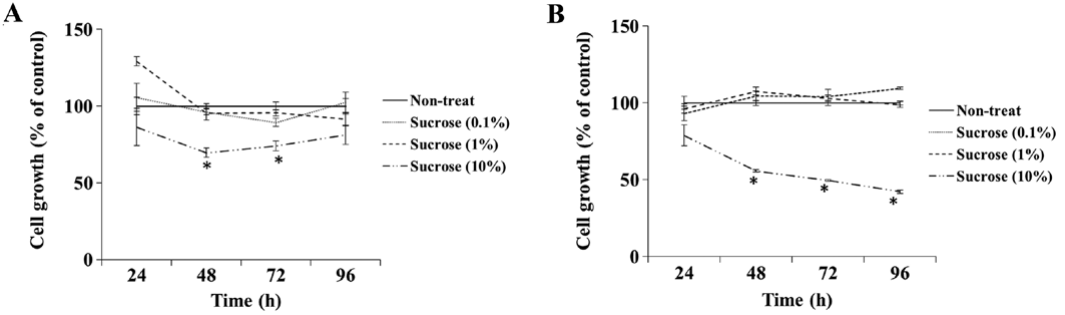
Cytotoxic effects of the different concentrations of sucrose on the proliferation of CRC cells. The suitable non-cytotoxic concentrations of sucrose for treating CRC cells with maple syrup were determined. (A) DLD-1 and (B) SW480 cells cultured in medium containing 10% sucrose exhibited decreased growth rates when compared with those in medium with lower concentrations of sucrose at 48 and 72 h and 48, 72 and 96 h, respectively. ^*^P<0.01 vs. the controls.

To examine the cytotoxic effect of high concentrations of sucrose on DLD-1 and SW480 cells, we assessed the cell growth rate when cells were grown in culture medium containing the control sucrose solution at a concentration of 0.1–10% (v/v). The growth rate of the DLD-1 cells cultured in the medium containing 10% sucrose solution was significantly inhibited at 48 and 72 h compared with the rate in the non-treated cells, whereas other concentrations of sucrose did not affect cell growth (Fig. 1A). The 10% sucrose solution also significantly inhibited the cell growth rate of SW480 cells at 48, 72 and 96 h (Fig. 1B). Thus, we determined the dose of maple syrup and sucrose solution as 1% since sucrose did not show cytotoxicity in the following experiments.

### Effect of maple syrup on the cell growth of CRC cells

**Figure 2:**
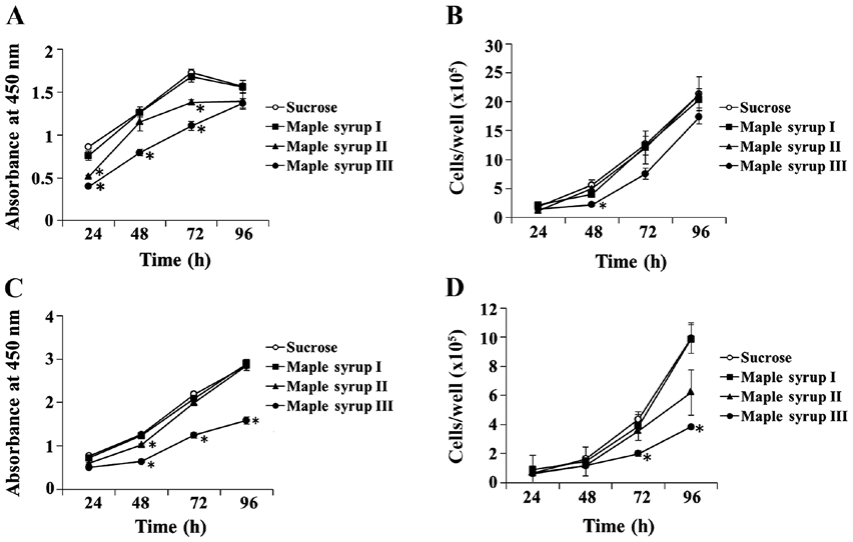
Effect of different maple syrup samples on the proliferation of CRC cells. (A) DLD-1 cells cultured in medium containing maple syrup showed decreased growth rates when compared with those grown in medium containing sucrose after 24 (maple syrup II and maple syrup III), 48 (maple syrup III), and 72 h (maple syrup II and maple syrup III) (^*^P<0.01) based on the WST-8 assay, and (B) at 48 h (maple syrup III) (^*^P<0.01) based on the cell counting method. (C) SW480 cells cultured in medium containing maple syrup showed decreased growth rates when compared with those grown in medium containing sucrose at 48 (maple syrup II and maple syrup III), 72 (maple syrup III), and 96 h (maple syrup III) (^*^P<0.01) based on the WST-8 assay, and (D) at 72 (maple syrup III) and 96 h (maple syrup III) (^*^P<0.01) based on the cell counting method.

We determined whether maple syrup had any effects on the growth of CRC cells. Administration of maple syrup significantly inhibited the cell growth of the DLD-1 cells at 24 h (maple syrup II and maple syrup III), 48 h (maple syrup III) and 72 h (maple syrup II and maple syrup III) using the WST-8 assay (P<0.01, Fig. 2A), and at 48 h (maple syrup III) using the cell counting method (P<0.01, Fig. 2B). Administration of maple syrup also inhibited the cell growth of the SW480 cells at 48 h (maple syrup II and maple syrup III), 72 h (maple syrup III), and 96 h (maple syrup III) using the WST-8 assay (P<0.01, Fig. 2C), and at 72 h (maple syrup III) and 96 h (maple syrup III) using the cell counting method (P<0.01, Fig. 2D).

### Effect of maple syrup on the cell growth of normal human colon epithelium cells

**Figure 3:**
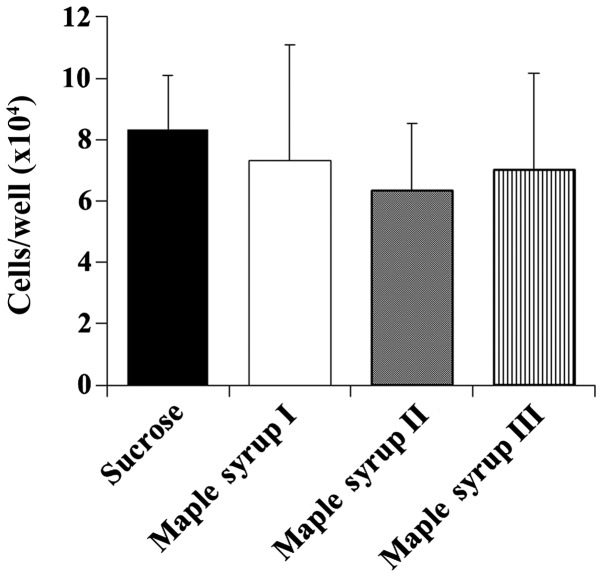
Effect of maple syrup on the proliferation of normal colonic epithelial cells. Maple syrup had no effect on the proliferation of normal colonic epithelial cells.

Next, we determined the effect of maple syrup on the cell growth of CCD 841 CoN cells to determine whether maple syrup has any effects on the growth of normal colon cells. Maple syrup administration had no effect on the growth of the CCD 841 CoN cells at 72 h despite that the growth of the CRC cells was inhibited (Fig. 3).

### Effect of maple syrup on cell migration and invasion

**Figure 4:**
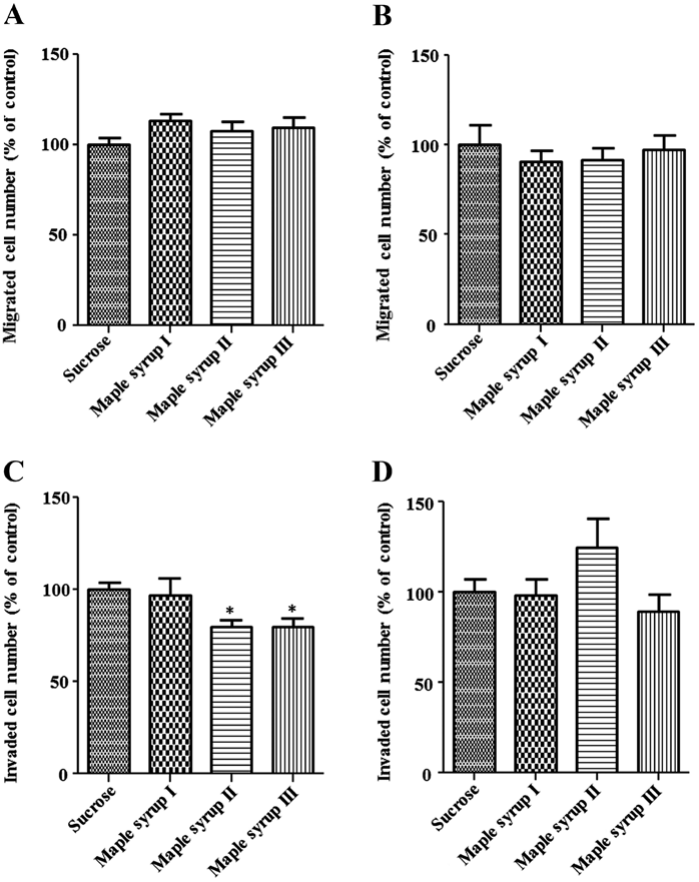
Effect of maple syrup on the migration and invasion of CRC cells. Cell migration and invasion assays were performed using a modified Boyden chamber method. (A and B) Maple syrup had no effect on the cell migration of either DLD-1 or SW480 cells. (C) Maple syrup II and maple syrup III significantly inhibited the cell invasion of DLD-1 cells (^*^P<0.01). (D) Maple syrup III tended to inhibit the cell invasion of SW480 cells (P=0.0779).

Next, we examined the migration and invasion of CRC cells using the modified Boyden chamber method. The number of cells that migrated from the inside of the chamber to the outside was not significantly affected by maple syrup in the DLD-1 and SW480 cells (Fig. 4A and B). However, an invasion assay using a Matrigel-coated chamber showed that the number of DLD-1 cells invading the Matrigel was significantly inhibited by maple syrup II and maple syrup III (P<0.01, Fig. 4C), and the number of SW480 cells invading the Matrigel tended to be inhibited by maple syrup III (P=0.0779, Fig. 4D)

### Effect of maple syrup on signaling pathways in CRC cells

**Figure 5:**
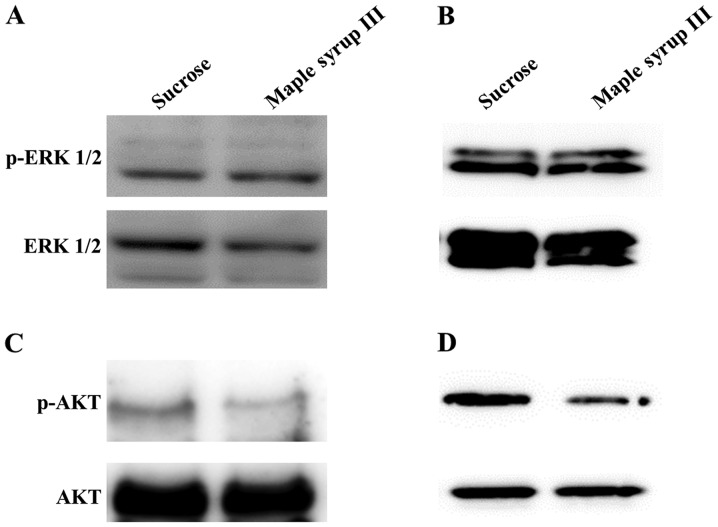
Effect of maple syrup on the ERK and AKT signaling pathways. Maple syrup had no effect on ERK 1/2 phosphorylation in the (A) DLD-1 or (B) SW480 cells. Maple syrup (III) inhibited AKT phosphorylation in the (C) DLD-1 and (D) SW480 cells.

To determine the signaling pathway that is affected by maple syrup, we examined the phosphorylation of ERK and AKT, which play important roles in cell proliferation and invasion in CRC cells, after administration of sucrose solution or maple syrup III. Administration of maple syrup III did not affect the phosphorylation of ERK (Fig. 5A and B). In contrast, we found that administration of maple syrup III clearly inhibited AKT phosphorylation (Fig. 5C and D).

## Discussion

In the present study, we examined the effect of different types of maple syrup on two different CRC cell lines. First, we examined whether the high concentration of sucrose in maple syrup affects CRC cell growth in order to clarify the anti-proliferative effect of maple syrup against CRC cells. High concentrations of sucrose significantly inhibited the growth of the CRC cell lines (Fig. 1). This result suggests that the high concentration (10%) of sucrose in maple syrup has cytotoxic effects due to high osmotic pressure. Previously, maple syrup was reported to inhibit the growth of prostate (74%), lung (63%), breast (45%) and colorectal (37% inhibition) cancer cell lines (19). However, these cancer cell lines were grown in culture medium containing 5% (v/v) maple syrup. Therefore, we were concerned that the growth of the cells might have been affected by high osmotic pressure due to the high concentration of sucrose. Thus, we estimated the dose of maple syrup that would not have cytotoxic effects for subsequent experiments.

Under these conditions, CRC cells grown in culture medium containing maple syrup had a decreased growth rate compared with those grown in culture medium containing sucrose, using both the WST-8 assay and cell counting method to determine growth rates (Fig. 2). Notably, the anti-proliferative effect of maple syrup increased as the color of the maple syrup became darker. We also examined the effect of maple syrup on the cell growth of normal colonic epithelial cells; however, none of the maple syrups tested affected the growth of normal colonic epithelial cells (Fig. 3). These data suggest that maple syrup may effectively inhibit rapidly growing cells such as cancer cells.

Moreover, cell invasive activity was significantly inhibited by maple syrup II and maple syrup III in the DLD-1 cells, and tended to be inhibited by maple syrup III in the SW480 cells, whereas there was no effect on the cell migration of either DLD-1 or SW480 cells (Fig. 4). These data suggest that maple syrup might affect the expression or activation of enzymes, such as MMP-2 and MMP-9, which play an important role in cell invasion of the extracellular matrix, including the basement membrane. Although there is no report on the effect of maple syrup on cell invasion, a recent study reported that polyphenols such as epigallocatechin-3-gallate inhibit MMP-2 and MMP-9 expression (24–28). Therefore, the expression levels of MMP-2 and MMP-9 in CRC cells might be inhibited by polyphenols such as quebecol and ginnalins that are present in maple syrup.

We examined the phosphorylation status of ERK and AKT using maple syrup III, which was the most effective maple syrup in our study. AKT activation was clearly inhibited by administration of maple syrup III, whereas ERK activation was not affected (Fig. 5). These data suggest that the anti-proliferative effect of maple syrup might be due to apoptosis induction. In addition, inhibition of AKT phosphorylation has been correlated with the inhibition of cancer cell invasion by reducing MMP-2 and MMP-9 expression (29–31). A recent study reported that ginnalins A-C, which are polyphenols present in maple syrup, inhibited cell growth through cell cycle arrest that did not induce apoptosis (17). Therefore, there is a possibility that maple syrup contains other effective compounds in addition to polyphenols. Further studies are needed to identify the compounds responsible for the inhibition of cell growth and invasion observed through the suppression of the AKT signaling pathway after the administration of maple syrup. In preparation for future research, we have begun to identify compounds using the high molecular weight fraction (MW >10,000) of maple syrup that is related to the inhibition of cell growth by maple syrup III, which demonstrated the strongest inhibitory effect among the maple syrup types we tested.

In conclusion, maple syrup, which is a natural sweetener used throughout the world, inhibits CRC cell growth and invasion through suppression of the AKT signaling pathway. These findings suggest that maple syrup, particularly dark colored ones, might be suitable as phytomedicines, which have fewer adverse effects than traditional chemotherapy for CRC treatment.

## Figures and Tables

**Table I:** Relative color and sucrose concentrations of the different maple syrup samples.

	Color	Sucrose (g/100 ml)
Maple syrup I	Slightly golden	60.01
Maple syrup II	Amber	62.89
Maple syrup III	Very dark brown	54.72
